# Pre-Injury Multi-Dimensional Health and 6-Month Outcome After Geriatric Traumatic Brain Injury

**DOI:** 10.1093/geroni/igaf122.2193

**Published:** 2025-12-31

**Authors:** Roy Tzemah-Shahar, Yael Rosen-Lang, Ava M Puccio, Esther L Yuh, Michele Diaz Nelson, Kristine Yaffe, Raquel C Gardner

**Affiliations:** University of Haifa, Haifa, HaZafon, Israel; Sheba Medical Center, Ramat Gan, Tel Aviv, Israel; University of Pittsburgh, Pittsburgh, Pennsylvania, United States; University of California, San Francisco, San Francisco, California, United States; University of California, San Francisco, San Francisco, California, United States; University of California, San Francisco, San Francisco, California, United States; Sheba Medical Center, Ramat Gan, Tel Aviv, Israel

## Abstract

Older adults with traumatic brain injury (oaTBI) are, on average, at greater risk for morbidity and mortality compared to younger adults. Most existing models predicts recovery measured by Glasgow Outcome Status Extended (GOSE, scored 1-8 while 1 means death and 8 returning to pre-injury state) based primarily on age and injury severity, and may not be optimized for oaTBI. In this study we tested the performance of four existing models developed to predict GOSE< 5 (unfavorable outcome, severe disability or worse) 6-month following the injury), in oaTBI cases. We additionally quantified the added prognostic value of pre-injury multi-dimensional health metrics: activities of daily living, comorbidity burden, cognitive status, and frailty. The analytic cohort included N = 112 participants from the TRACK-GERI study, a 2-center prospective cohort study of adults aged 65 years and older presenting to Level 1 Trauma Centers with acute TBI who undergo head CT (95% with Glasgow Coma Scale 13-15, 33% with ICU admission). The CRASH Basic model had the highest accuracy for discriminating GOSE< 5 (AUC 0.74, 95% CI 0.64,0.83). Incorporating pre-injury characteristics improved the model’s ability to discriminate favorable vs. unfavorable outcome, however the change was statistically insignificant. Frailty, measured using the Groningen Frailty Indicator, was an independent predictor of poor outcome (OR 1.30, 95% CI 1.06,1.61). These findings demonstrate the role pre-injury characteristics play in oaTBI recovery process; integrating such factors in new outcome prediction models may improve clinical decision-making and individualized outcome prediction.

